# Sonographic Demonstration of Intracranial Hemorrhage in a Fetus with Hydrops Fetalis due to Rh Alloimmunization after Intrauterine Intravascular Transfusion: A Case Report and Review of the Literature

**DOI:** 10.1155/2018/8412139

**Published:** 2018-03-26

**Authors:** Rauf Melekoglu, Ebru Celik, Hasim Kural

**Affiliations:** ^1^Department of Obstetrics and Gynecology, Faculty of Medicine, The University of Inonu, 44280 Malatya, Turkey; ^2^Department of Obstetrics and Gynecology, Faculty of Medicine, The University of Koc, 34010 İstanbul, Turkey

## Abstract

Intrauterine transfusion is the most common and successful intrauterine procedure for the treatment of fetal anemia due to red cell alloimmunization. Fetal intracranial hemorrhage is a very rare complication of intrauterine transfusion in patients with Rh(D) alloimmunization and it has been demonstrated only in a few case reports in the literature. Herein, we described a case of grade IV intraventricular hemorrhage that was diagnosed following the first intrauterine transfusion and reviewed the literature about the fetal intracranial hemorrhage that occurred after intrauterine intravascular transfusion procedure.

## 1. Introduction

Intrauterine transfusion (IUT) is the most common and successful intrauterine procedure for the treatment of fetal anemia due to red cell alloimmunization [1]. The beneficial effect of in utero therapy on perinatal survival has been demonstrated clearly in several observational studies [2, 3]. Despite the dramatic decrease in the IUT requirement due to the widespread use of prophylactic Rh(D) immune globulin, the procedure continues to be a gold standard for treatment of severe fetal anemia [4]. While intrauterine intravascular transfusion has remarkable effect on the treatment of fetal red blood cell alloimmunization, the total procedure-related complication rate has been reported approximately 3.1 percent and commonly indicated as fetal death, neonatal death, emergency cesarean delivery, infection, and premature rupture of membranes [5]. Fetal brain injury is a very rare complication of IUT that has been demonstrated only in a few case reports in the literature [6]. To the best of our knowledge, the literature does not include any cases of a grade IV intraventricular hemorrhage due to IUT.

Herein, we described a case of grade IV intraventricular hemorrhage that was diagnosed following the first IUT and reviewed the literature about the fetal intracranial hemorrhage that occurred after intrauterine intravascular transfusion procedure in patients with Rh(D) alloimmunization. We searched PubMed, Scopus, Embase, and Google Scholar databases using the keywords Rh isoimmunization “OR” intrauterine transfusion “AND” intracranial hemorrhage “OR” brain injury “OR” brain damage. We found only two papers that define three cases of intracranial hemorrhage associated with intrauterine transfusion due to Rh alloimmunization. The initial platelet value was not noted in the third case. Author, case number, patient's age, gestational age, pretransfusion hemoglobin value, pretransfusion platelet value, neurosonogram after the first intrauterine transfusion, and the outcome were summarized in Table 1. In this paper, we have compared our case with the other three cases we have found in the literature.

## 2. Case Presentation

**Figure 1:**
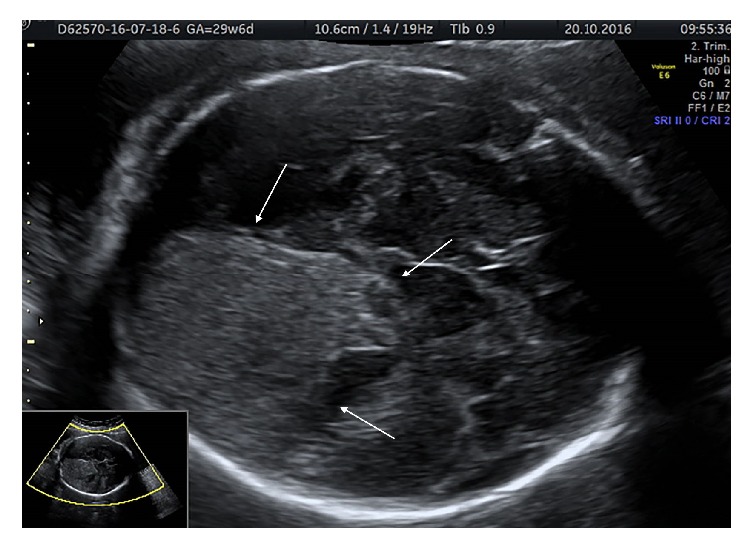
Axial view of the fetal head after intrauterine transfusion procedure. A large echogenic collection involving right lateral ventricle and extending to the surrounding parenchyma that suggests grade IV intraventricular hemorrhage.

A 34-year-old woman, gravida 3, para 2, with a history of an intrauterine death at 32 weeks of gestation due to hydrops fetalis as a result of Rh alloimmunization in the previous pregnancy was referred to our center at 13 weeks of gestations. There were no pathologic findings in her physical examination, laboratory findings, and obstetric ultrasonography. Gray-scale and color Doppler ultrasonography evaluation was performed to detect the findings of anemia and hydrops with a two-week interval starting from the 18th gestational week in the Prenatal Diagnosis and Treatment Unit by a protocol defined by Society for Maternal-Fetal Medicine for the pregnant women complicated with Rh alloimmunization [7]. Her obstetric follow-up was unremarkable until 29 weeks of gestation. At 29 weeks of gestation, mid-cerebral artery peak systolic velocity (MCA-PSV) was detected as 80.6 cm/sn [>1.5 multiple of the median (MoM)] in her obstetric Doppler ultrasonography that suggests fetal anemia with ascites, cardiomegaly, and pericardial effusion. An intrauterine intravascular transfusion was performed, and the hemoglobin concentration before the procedure was detected as 2.9 g/dL. 40 ml O Rh(D)-negative red blood cell that was freshly prepared and underwent irradiation and leukodepletion was transfused to the fetus via the umbilical vein in the portion of the umbilical cord near its insertion into the placenta by a 22-gauge needle with no complication. Posttransfusion hemoglobin was detected as 8.1 g/dl. The initial and posttransfusion platelet count was detected in normal range (154000/*μ*l and 163000/*μ*l, resp.). A few days later, ultrasound examination revealed the presence of an echogenic collection involving right lateral ventricle and extending to the surrounding cerebral parenchyma compatible with grade IV intraventricular hemorrhage (Figure 1). The couple was counseled, and they opted for the continuation of in utero therapy. The second IUT was scheduled after ten days. At 30 + 4 weeks of pregnancy, the second IUT was performed. The initial hemoglobin value was detected as 4.9 g/dl. Persistent fetal bradycardia was noted during the procedure, and an emergency cesarean section was performed. APGAR score 4/7, 1315 g, 43 cm male infant was delivered by cesarean section. Neonate was transferred to the neonatal intensive care unit. Exchange transfusion, phototherapy, and intravenous immunoglobulin treatment were applied. Postnatal cranial ultrasonography showed diffuse echogenicity extending from the inferior left caudate nucleus to the left ventricle that leads to left ventricular dilatation compatible with intraventricular grade IV hemorrhage. The intracranial hemorrhage was gradually regressed in the subsequent ultrasonographic examinations and completely disappeared at the end of the first month, and the neonate was discharged from the hospital after healing two months after birth. At the time of writing this paper, the baby was showing normal neurological development at 6 months.

## 3. Discussion

Intrauterine transfusion has been reported as the most successful fetal therapy procedure with 95% perinatal survival rate [8]. The overall survival rate after this antenatal treatment procedure varies with experience of center, development of fetal anemia before 20 weeks of gestation, and occurrence of fetal hydrops [9]. The presence of fetal hydrops during the first IUT reduces the success of the treatment. Lindenburg et al. reported the perinatal outcome of 491 fetuses who underwent 1422 intrauterine intravascular transfusion procedure during the antenatal period. They demonstrated that perinatal survival rate was 83% and 95% in hydropic and nonhydropic fetuses, respectively [10]. Although IUT contributes to the reduction of perinatal mortality, concerns about the neurological morbidities associated with this procedure have been considered only in a few studies [11]. Fetal intracranial hemorrhage as a short-term neurological morbidity was reported by Ghi et al. for the first time in 2003 [12]. They described four cases with intracranial hemorrhage related to the fetal anemia (two immune hydrops due to Rh D alloimmunization, two monochorionic twins complicated with the death of the cotwin). Consistently with the case currently reported, each of the cases related to Rh alloimmunization had very low initial hemoglobin values in the first IUT (1.2 g/dl and 1.6 g/dl, resp.). They suggested that disruption of intracranial vessels may be responsible in the pathophysiology of brain injury in severe anemic fetuses and noticed the importance of fetal neurosonography in pregnancies with severe anemia due to Rh alloimmunization undergoing IUT. In 2004, the same group reported multiplanar neurosonography results of seven consecutive hydropic fetuses undergoing intrauterine transfusion procedure due to Rh alloimmunization [13]. In addition to the previously reported two cases, they described a case of periventricular leukomalacia and a case of unilateral ventriculomegaly that was noticed after the first IUT. They speculated that hypoxia/ischemia and the hyperdynamic circulation in fetal anemia cause the brain vessel disruption that leads to intracranial hemorrhage. They also considered that altered coagulation due to IUT might be responsible for intracranial hemorrhage. In our case, the initial and posttransfusion platelet values were detected in the normal range (154000/*μ*l and 163000/*μ*l, resp.), and there was no sign of increasing bleeding time such as excessive bleeding from the umbilical cord after withdrawal of the needle. Furthermore, we hypothesized that preservative-anticoagulant system such as additive solution-1 (AS-1), AS-3, AS-5, citrate-phosphate-dextrose-adenine-1 (CPDA-1), citrate-phosphate-dextrose (CPD), and citrate-phosphate-dextrose-dextrose (CP2D) that were used in red blood cell preparation might predispose to intracranial hemorrhage by altering the coagulation system of the fetus. Thus, we proposed that removing these anticoagulants from the transfusion aliquots before intrauterine transfusion by centrifugation and volume reduction could have a beneficial effect on preventing the hemorrhagic complication of this treatment.

Simonazzi et al. demonstrated the risk of cerebellar damage in fetuses with severe anemia due to RhD alloimmunization after intrauterine intravascular transfusion procedure [14]. They reported three cases of intracranial hemorrhage involving cerebellum that two of them were previously reported by Ghi et al. In the third case, they performed first IUT at 22 weeks of gestation and after two weeks they noted suspicious cerebellar infarction in prenatal ultrasonography. In postnatal magnetic resonance imaging, bilateral cerebellar hemosiderin staining suggested prior hemorrhage. They emphasized that intracranial hemorrhage occurred at the infratentorial part of the brain particularly in intracerebellar hemispheres in all of the tree cases. Furthermore, in addition to hypoxia/ischemia, they also noticed the possible serious effect of sudden fluctuations in cerebral blood flow and arterial blood pressure (hyperdynamic circulation) on the intracranial hemorrhage. In our case, fetal anemia and hydrops were detected at the 28th week of gestation that was developed later compared with the other cases previously reported. Moreover, intracranial hemorrhage was identified in the right lateral ventricle and extending to the surrounding cerebral parenchyma compatible with grade IV intraventricular hemorrhage. Therefore, we considered that intracranial hemorrhage risk is not related to gestational week of the first IUT in the presence of Rh(D) alloimmunization and posttransfusion intracranial hemorrhage is not specific only the infratentorial region of the brain. Also it has been suggested that, in pregnancies complicated with the severe fetal hemolytic disease, an initial extremely low value of hematocrit (≤15%) should be increased gradually for the risk of fetal cardiovascular decompensation due to the acute changes in blood volume and viscosity. In such cases, the planning of the second procedure is recommended after 48 hours of the first transfusion to normalize the fetal hematocrit value [15, 16]. Consistently with the other cases reported previously, intracranial hemorrhage occurred after the first intrauterine intravascular transfusion. Consequently, we concluded that reduction of first transfusion volume might be beneficial to avoid sudden fluctuations in cerebral blood flow and arterial blood pressure.

In conclusion, in this study, a case of intracranial hemorrhage in a fetus with hydrops fetalis due to Rh alloimmunization after intrauterine intravascular transfusion has been presented and a comprehensive, up-to-date review has been performed. Although intracranial hemorrhage is a rare complication of IUT, clinicians should be aware of increased risk of brain damage in fetuses with Rh(D) alloimmunization undergoing this procedure. Detailed sonographic examination of the fetal central nervous system before and after the treatment should be performed in patients that are planning IUT. Also, communicating with blood bank to decrease the additive anticoagulant agents as minimal as possible in the preparation process of red blood cells and reduction of first transfusion volume to avoid sudden fluctuations in cerebral blood flow may be helpful to prevent this complication.

## Figures and Tables

**Table 1:** Summary of the reported fetal intracranial hemorrhage cases related to intrauterine transfusion due to Rh alloimmunization.

Authors	Case number	Maternal age	Gestational age	Pretransfusion hemoglobin value (g/dl)	Pretransfusion platelet value (/*μ*l)	Neurosonogram after the first intrauterine transfusion	Outcome
Ghi et al. 2004	Case 1	30	20	1.2	168000	Intraventricular and cerebellar hemorrhage	Termination of pregnancy. Pathological confirmation of cerebellar hemorrhage
	Case 2	25	23	1.6	177000	Cerebellar hemorrhage	Progressive hypoplasia of one cerebellar hemisphere. Delivery at 34 weeks after six IUTs. Normal neurological development at 2 years

Simonazzi et al. 2016	Case 3	32	22	4	-	Suspicious cerebellar infarction	Hemosiderin staining in the cerebellum bilaterally, reflecting prior hemorrhage in postnatal brain MRI. Normal neurological development at 14 months

Current study	Case 4	34	28	2.9	154000	Echogenic collection in the right lateral ventricle and extending to the surrounding cerebral parenchyma compatible with grade IV intraventricular hemorrhage	Diffuse echogenicity extending from the inferior left caudate nucleus to the left ventricle that leads left ventricular dilatation (intraventricular grade IV hemorrhage). Normal neurological development at 6 months
